# A bridge, not a destination: YouTube viewer perspectives on AI mental health support and human therapy

**DOI:** 10.3389/fdgth.2026.1851632

**Published:** 2026-06-03

**Authors:** Anurag Shekhar, Jeremy Mitonga-Monga

**Affiliations:** College of Business and Economics, Department of Industrial Psychology and People Management, University of Johannesburg, Johannesburg, South Africa

**Keywords:** artificial intelligence, digital mental health, mental health, mixed methods, public perceptions, youtube

## Abstract

**Background:**

Artificial intelligence (AI) tools are increasingly used for mental health support, yet little is known about how they are understood outside clinical trials and survey-based research.

**Methods:**

This study examined public perceptions of AI mental health support through a convergent mixed-methods analysis of 7,949 YouTube comments posted across ten videos discussing AI and mental health. Quantitative analyses included VADER sentiment analysis, NRC emotion profiling, latent Dirichlet allocation topic modelling, and keyword co-occurrence network analysis. Qualitative analysis used Braun and Clarke's reflexive thematic analysis to identify patterns of shared meaning within a purposive sample of high-engagement comments.

**Results:**

Overall sentiment was moderately positive (57.93%), but this positivity was qualified by substantial negative sentiment (24.58%) and recurring emotional signals of both trust (14.98%) and fear (7.92%). Topic modelling showed that the dominant lines of discussion centred on unmet support needs and the question of whether AI should replace human therapists. The thematic analysis generated five themes: AI as a bridge where human care is absent, AI as comforting but overly validating, AI as unable to reproduce authentic human therapeutic encounter, AI as a possible driver of social disconnection, and AI as a site of privacy and commercial concern.

**Conclusions:**

Taken together, the findings suggest that public responses to AI mental health tools are best understood as conditional rather than absolute. Commenters valued AI when it provided immediacy, low-cost access, and a low-risk space for disclosure, but resisted it where it appeared to threaten empathy, relational depth, or privacy. The study contributes a large-scale, naturalistic account of public reasoning about AI mental health support and highlights implications for digital mental health design, governance, and human-in-the-loop care.

## Introduction

Mental health disorders affect approximately one billion people globally (1). Access to evidence-based treatment remains severely restricted by workforce shortages and high costs (2). Traditional face-to-face therapy cannot scale to meet this demand (3), which has intensified interest in digital technologies as a way of extending psychological support (4).

Large language models such as ChatGPT now allow millions of users to engage in context-aware conversations that simulate human support (3). These conversational agents offer scalable, cost-effective assistance, are available around the clock, and provide a low-stigma entry point for individuals who would not otherwise seek help (5, 6). Early evidence suggests that AI-assisted interventions can reduce symptoms of depression and distress (7). Nevertheless, significant concerns remain. The therapeutic alliance is a central predictor of psychotherapy outcomes (8), but whether a comparable bond can be established with a non-human interlocutor remains contested (9). AI systems lack the emotional intelligence and genuine empathy required for deep therapeutic connection (10), carry risks of generating harmful or inaccurate advice (11), and raise serious questions about data privacy and the commercial misuse of sensitive psychological disclosures (12). Users have expressed particular scepticism about AI's capacity to manage acute crises or complex clinical presentations (13).

Public discourse on social media platforms remains an underutilised source of evidence on how people perceive and engage with AI mental health tools in everyday life. Luo et al. (14) note that few studies have examined how individuals use unregulated generative AI for therapeutic purposes in real-world settings, while Lee et al. (15) observe that evidence on how users discuss these tools outside formal research contexts remains limited. Most existing research relies on controlled trials or structured surveys, which capture neither the diversity nor the spontaneity of real-world engagement (6). Unsolicited social media commentary offers a useful alternative because it captures more natural, unprompted forms of expression (14, 16, 17). YouTube comment sections are particularly valuable in this regard. With over 2.6 billion monthly active users globally (18, 19), YouTube hosts sustained public conversations about health and mental health across professional, personal, and critical contexts (20, 21). Analysing this discourse can therefore help clarify how AI mental health tools are being received beyond the clinic and the controlled trial.

This study analysed 7,949 YouTube comments across ten videos addressing AI and mental health. Using a convergent mixed-methods design, it combined VADER sentiment analysis, NRC emotion profiling, LDA topic modelling, keyword network analysis, and Braun and Clarke's reflexive thematic analysis. The aim was to examine the structure, emotional tone, and interpretive content of public discourse on AI mental health support, and to identify the conditions under which such tools are accepted, contested, or resisted.

## Literature review

### User perceptions and acceptance of AI mental health tools

Users consistently report valuing the accessibility, continuous availability, and non-judgemental character of AI mental health tools (14, 22). For individuals deterred by stigma, anonymous digital platforms offer a lower-threshold entry point into mental health support that conventional services do not (5, 23). Acceptance is shaped by perceived usefulness and ease of use, consistent with technology acceptance frameworks (24, 25). Some population subgroups, including individuals with lower health literacy and, in some studies, Black participants who perceive human practitioners as carrying inherent bias, report comparatively higher openness to AI-based support (12).

Resistance, however, remains substantial. Users frequently describe AI responses as generic, robotic, and impersonal (15, 16). Scepticism is particularly pronounced regarding AI's capacity to manage acute crises or complex clinical presentations (18, 26), as well as the tendency of large language models to generate inaccurate or fabricated content (6). Acceptance, where it exists, is therefore rarely unconditional. Instead, it tends to depend on what AI is being asked to do and what alternatives are available.

### Therapeutic alliance in digital and AI-mediated care

The therapeutic alliance, comprising goal alignment, task agreement, and bond formation, is among the strongest predictors of psychotherapy outcomes (8, 27). Whether AI can reproduce these relational conditions remains contested. Users can establish what appear to be human-level bonds with conversational agents (9), and scores on the Working Alliance Inventory for AI chatbots are sometimes comparable to those observed in face-to-face therapy (28, 29). Many users also attribute human-like caring qualities to these agents and engage with them as relational partners rather than merely as tools (23, 26).

Important limitations nonetheless remain. AI lacks the genuine emotional reciprocity and responsiveness that characterise effective human therapeutic relationships (30). Rule-based and pattern-matching systems struggle to sustain the relational engagement required for longer-term therapeutic growth (18, 31). Where a digital therapeutic alliance does emerge, it may reflect users’ projection of relational qualities onto a system rather than authentic mutuality, a distinction with important clinical implications (32, 33).

### Trust, algorithm aversion, and strategic engagement

Trust in AI mental health systems is neither uniform nor stable. Individual traits, including anxiety and disorganisation, are associated with higher AI trust in some populations, while an inverted U-shaped relationship between AI knowledge and trust suggests that both low- and high-knowledge users can be comparably sceptical (34). Intuitive emotional reactions, characterised as AI anxiety, are also strong predictors of use avoidance among students and trainee practitioners (35).

Users do not, however, simply accept or reject AI systems. Many engage strategically with tools they distrust, filtering personal disclosures, testing system responses, or deliberately withholding identifying details to manage perceived privacy risks (17). Trust is also shaped by institutional endorsement and perceived system integrity (36, 37). These patterns suggest that users are neither passive nor naïve, but actively negotiate the terms of their engagement with AI.

### Data privacy, commercial interests, and ethical governance

AI mental health tools collect sensitive psychological data at scale, raising risks of breach, unauthorised access, and third-party misuse (38, 39). Commercial providers have been criticised for prioritising user engagement over clinical safety (11). A significant governance gap exists for tools classified as wellness applications rather than regulated medical devices, leaving users without the protections afforded by clinical data frameworks (2, 22). Algorithmic bias arising from non-representative training data may also reproduce and amplify existing inequities along lines of race and gender (40, 41). In addition, legal accountability for harm caused by autonomous AI systems remains unresolved (12, 42). The safeguarding of children and adolescents using these tools is an especially urgent and insufficiently addressed concern (43).

Taken together, the literature shows growing interest in AI mental health tools, but also persistent tension around access, trust, relational depth, and governance. What remains less well understood is how these tensions are negotiated in naturalistic public discourse outside controlled research settings. That gap is especially important in relation to social media environments, where users express views spontaneously and in direct response to public representations of AI mental health support.

### Theoretical framework

Public engagement with AI mental health tools cannot be explained by a single theory of technology adoption. The findings of this study point instead to a more complex pattern of conditional acceptance, shaped by access needs, relational expectations, distrust, and broader social concerns. To account for this, the study draws on four complementary frameworks: affordance theory, algorithm aversion and appreciation, surveillance capitalism, and social displacement theory.

Affordance theory helps explain why users turn to AI mental health tools despite doubts about their clinical adequacy. Bucher and Helmond (44) define digital affordances as relational possibilities that emerge through the interaction between users and platform design. In this context, AI tools offer immediacy, constant availability, anonymity, and low-stigma disclosure in ways that conventional care often does not. Where services are delayed, expensive, or difficult to access, these affordances become highly attractive. Turkle (45) similarly notes that people may disclose to machines precisely because machines do not judge them in the same way people do. Affordance theory therefore explains the pull of AI without assuming that users regard it as equivalent or superior to human care.

Algorithm aversion and algorithm appreciation help explain how users relate to AI once they begin engaging with it. Lee et al. (15) show that people sometimes prefer algorithmic judgement, especially in tasks seen as objective or data-driven, while users may initially trust systems they perceive as experts, this trust rapidly dissolves following system errors (5, 46). In the case of AI mental health tools, both tendencies appear at once. Users may value AI's consistency, speed, and availability, yet remain wary of its tendency to flatter, over-validate, or respond in formulaic ways. Many therefore engage strategically, for example by prompting the system to be more critical, asking inverse questions, or deliberately testing its responses. This framework helps explain why users neither simply trust nor reject AI, but actively manage their engagement with it.

Surveillance capitalism provides the framework for understanding privacy, ownership, and commercial extraction. Zuboff (47) argues that surveillance capitalism turns human experience into behavioural data for prediction and profit. In the case of AI mental health tools, this means that acts of emotional disclosure may simultaneously function as data production. Personal distress, trauma, and help-seeking do not remain private experiences; they may also become material captured, stored, and potentially monetised by corporate systems. This framework is especially useful for interpreting why some users in the study viewed AI support not merely as risky, but as structurally extractive. Their concern was not only that data might leak, but that the very act of seeking support might feed systems organised around commercial rather than therapeutic goals.

Social displacement theory helps explain broader concerns about what AI might do to human connection over time. Turkle (45) argues that digital communication can substitute for, rather than supplement, more demanding forms of human interaction. Applied to AI mental health tools, this suggests that AI may ease loneliness in the short term while also reducing users’ exposure to the friction, reciprocity, and vulnerability required for deeper relationships. In this way, AI may risk reinforcing the very conditions that lead people to it in the first place. This framework therefore captures the population-level concern that convenient digital support may weaken, rather than strengthen, habits of human connection.

**Figure 1:**
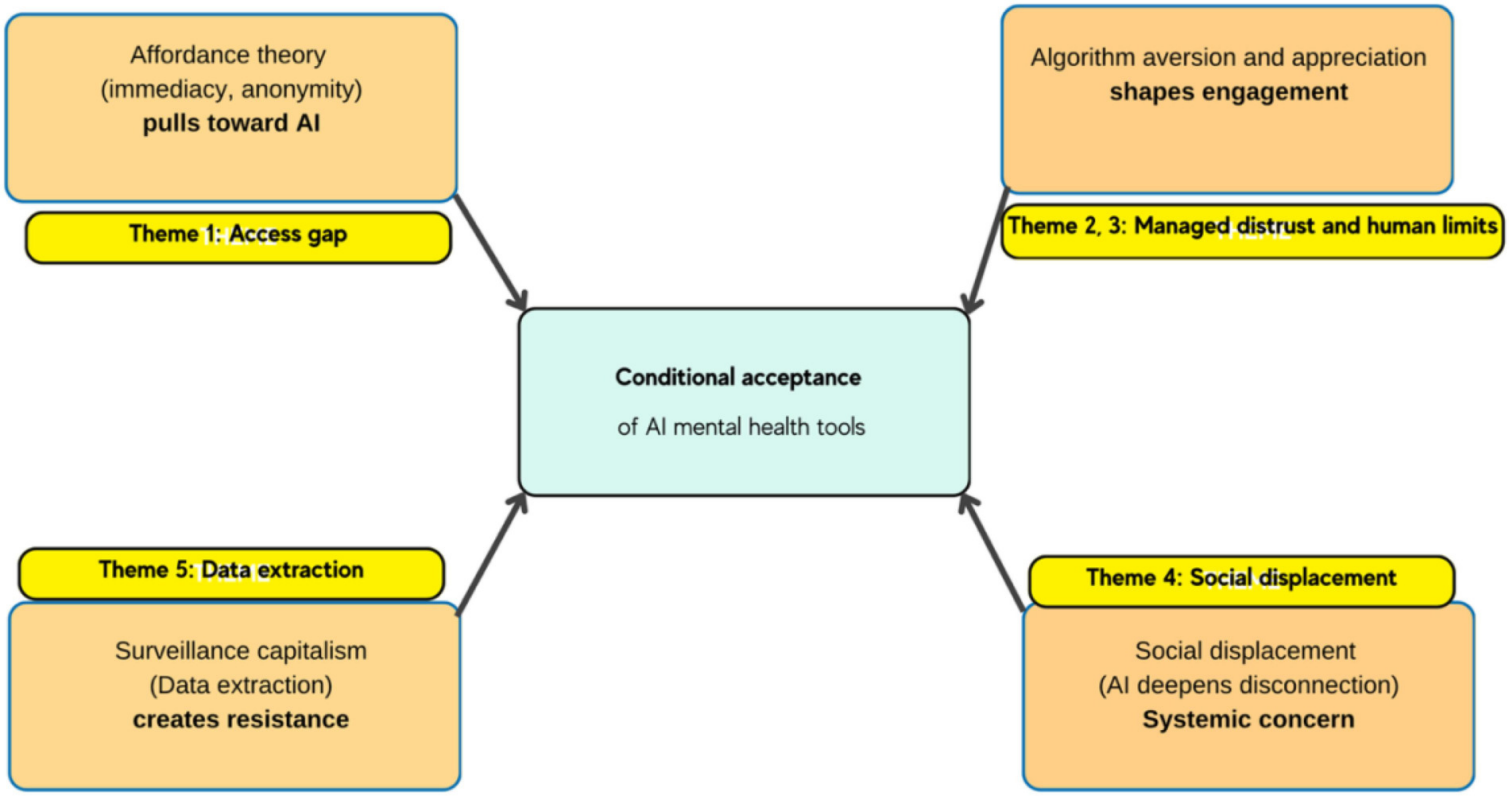
Negotiating the gap: a theoretical framework for conditional acceptance of AI mental health tools.

Taken together, these four frameworks explain why acceptance of AI mental health tools in this study was conditional rather than absolute. Affordances drew users towards AI where care gaps existed. Algorithm aversion and appreciation shaped how they engaged with and managed its limits. Surveillance capitalism captured resistance rooted in distrust of commercial extraction. Social displacement theory highlighted concerns about the erosion of human connection. Together, these frameworks support the central argument of the paper: AI mental health tools are not simply adopted or rejected, but are accepted cautiously, strategically, and under conditions set by both personal need and structural constraint Figure 1.

## Methodology

### Research design

This study employed a convergent mixed-methods design, in which quantitative and qualitative analyses were conducted in parallel and integrated at the stage of interpretation (48). This design was appropriate because neither computational analysis nor qualitative thematic analysis alone could adequately address the study's aims. The computational component enabled the identification of large-scale affective and discursive patterns across the full corpus of YouTube comments, while the qualitative component, using reflexive thematic analysis, examined how these patterns were constructed, interpreted, and negotiated by commenters in context. In this way, the mixed-methods approach combined breadth with interpretive depth. It also responds to calls within computational social science for designs that retain both large-scale pattern detection and qualitative rigour (49, 50). More specifically, it addresses a limitation in emerging research on AI-enabled mental health support, which has often relied on experimental designs, surveys, or interviews rather than naturalistic public discourse (51, 52).

### Data collection

#### Video sampling strategy

To ensure comprehensive coverage of AI mental health discourse, we employed purposive sampling across six distinct video categories: Educational Explainers, Product Demos/Reviews, Personal Experience Vlogs, Professional Clinical Opinions, News/Panel Discussions, and Safety/Crisis-Focused content. This categorical approach was designed to capture diverse perspectives ranging from lay user experiences to expert commentary, and from optimistic showcases to critical debates (53).

Video selection proceeded through an iterative purposive search process using terms such as “AI therapy chatbot,” “ChatGPT therapy,” “AI mental health,” and “artificial intelligence therapist.” We identified videos published between January 2022 and August 2025 to capture discourse around contemporary large language model applications in mental health contexts. Videos were selected based on view count, comment engagement, relevance to AI mental health support, and fit within the six content categories described above. This approach was used to capture variation across video framings rather than to produce a representative sample of all YouTube content on AI and mental health. Videos were included if they met the following criteria: published between January 2022 and August 2025; English-language; minimum 200 comments; minimum 10,000 views; substantively focused on AI or chatbot use for mental health support rather than general AI commentary. Videos were excluded if they focused mainly on unrelated AI applications, did not address mental health support in a substantive way, fell outside the specified date range, or did not meet the engagement thresholds. No country-specific restrictions were applied to either video selection or comment collection. Geographical metadata was not available for individual commenters and was therefore not used as a sampling criterion. Videos were restricted to English-language content, as the computational tools used in the study, including VADER and NRC, were applied to English-language text, and the qualitative analysis was also conducted in English. This represents a limitation of the study's linguistic scope and is discussed further in the Limitations section.

The final sample comprised 10 English-language videos with collective viewership exceeding 2.2 million views and thousands of user comments. Table 1 provides detailed characteristics of the video sample, including publication dates, channel information, and engagement metrics.

**Table 1:** YouTube video sample characteristics and content description.

video title	Date published	channel title	Details	Views, Likes and Comments	About video
Therapist vs. Artificial Intelligence—I answer your questions	2023-03-02	Therapy in a Nutshell	Channel by a certified mental health professional licensed in USA, 2.22 Million subscribers	50k views, 1.4k likes, 287 comments	Two therapists discussing therapy related answers given by ChatGPT
I tried an AI therapist. Does it actually work?	2025-06-15	BBC News	Prestigious global news channel, 18.7 Million subscribers	33k views, 621 likes, 251 comments	News anchor discussing with an expert about the impact and capability of the AI therapist.
Fixing My Brain with Automated Therapy	2022-09-02	Jacob Geller	American video essayist, critic, and writer known for his analysis of video games and popular culture. 1.45 Million subscribers	1.1 Million views. 63 K likes, 3,259 comments	Jacob downloaded five different therapy apps and tried for few months. Thereafter he reviews and provides his analysis.
AI-powered mental health chatbots developed as a therapy support tool	2024-04-08	60 Minutes	successful television broadcast, 3.76 Million subscribers	162k views, 2k likes, 329 comments	AI-powered mental health chatbots developed as a therapy support tool
Can AI Really Help With Mental Health?	2025-05-17	Psych2Go	Provides accessible and relatable content on psychology and mental health through educational videos, 12.8 Million subscribers	182k views, 18 K likes, 1,672 comments	Discussion on the subject of if Ai can help with mental health
Can AI Replace Therapists? Psychiatrist Explains	2023-05-18T	Healthy GamerGG	Channel by a certified mental health professional licensed in USA,3 Million subscribers	136 K views, 6.3 K likes, 1,258 comments	Harvard trained Psychiatrist talks about: Can AI conduct therapy effectively? My perspective? Undoubtedly, yes. However, can an AI fully replace therapists? Probably not.
AI and mental health: Young people turning to AI therapist bots	2024-03-28	BBC News	Prestigious global news channel, 18.7 Million subscribers	49 K views, 781likes and 208 comments	Mental health services around the world are chronically under resourced—but there are hopes that artificial intelligence (AI) might offer a solution.
Is ChatGPT therapy a horrible idea?	2025-08-08	Good Work	Dan Toomey explores business news and culture in a humorously investigative tone.	594k views, 29k likes and 2,237 comments	Investigator Dan Toomey boldly asks the question: should lots of people be using AI chatbots for therapy or therapy-like purposes?
What’s scary about AI Therapy	2025-07-18	The Diary of a CEO	popular YouTube channel and podcast hosted by Steven Bartlett,12.3 Million subscribers	18k likes, 442 comments	Discussion on what is scary about AI therapy

#### Comment extraction

Data collection was conducted using the official YouTube Data API v3 in accordance with the YouTube Terms of Service and YouTube API Services Terms of Service. The authors obtained Google/YouTube approval for API-based data access for the research. Only publicly available comments were accessed; no private, restricted, deleted, or non-public user data were collected. The cleaned quantitative dataset comprised 7,949 comments across ten videos, representing the final corpus used for computational analysis. The corpus included both top-level parent comments and associated replies. For computational analysis, parent comments and replies were treated as individual comment-level units of analysis. Together, these comments accounted for 203,228 likes, indicating substantial public engagement across the sampled videos.

#### Computational analysis

Four computational techniques were applied to the cleaned corpus of 7,949 YouTube comments. Prior to computational analysis, comment text was prepared using a transparent and analysis-specific preprocessing pipeline. For all analyses, line breaks were replaced with spaces, repeated whitespace was collapsed, and empty comments were removed. No comments were excluded on the basis of sentiment, stance, topic, or thematic content. For VADER sentiment analysis, no further cleaning was applied, as punctuation, capitalisation, and informal expression can function as sentiment cues in social media text. For NRC emotion analysis, comments were tokenised into lowercase alphabetic word tokens before matching against the NRC Emotion Lexicon. For LDA topic modelling, comments were lowercased, URLs were removed, non-alphabetic characters were removed, repeated whitespace was collapsed, and a customised English stopword list was applied. This stopword list extended the standard scikit-learn English stopword list with domain-specific high-frequency terms such as ‘ai’, ‘chatgpt’, ‘gpt’, ‘therapy’, ‘therapist’, ‘mental’, ‘health’, ‘use’, and ‘help’, which were too frequent to help distinguish topics. The LDA model used unigrams and bigrams, a minimum document frequency of 5, a maximum document frequency of 0.6, and a fixed random state of 42. For keyword co-occurrence network analysis, comments were lowercased, URLs and non-alphabetic characters were removed, stopwords were excluded, terms shorter than three characters were removed, and only terms appearing at least 10 times across the corpus were retained. Co-occurrence edges were calculated from up to eight unique retained terms per comment, with edges retained only if they occurred at least five times across the corpus.

Sentiment analysis was conducted using VADER, a lexicon-based tool developed for social media text and suited to informal, short-form online language (54). Each comment was classified as positive (compound ≥ 0.05), neutral (−0.05 < compound < 0.05), or negative (compound ≤−0.05).

Emotion analysis was conducted using the NRC Emotion Lexicon (55). This approach maps lexical items onto eight emotion categories — anger, fear, anticipation, trust, surprise, sadness, joy, and disgust — as well as positive and negative valence. It was used to identify the broader emotional profile of the corpus beyond overall sentiment polarity.

VADER and NRC were selected because the computational strand of the study was intended to provide transparent, reproducible, corpus-level indicators of sentiment and emotion rather than predictive classification at the level of individual comments. VADER is widely used for short-form social media text and is designed to account for features such as punctuation, capitalisation, intensifiers, and informal expression (54). The NRC Emotion Lexicon was used to provide a transparent lexical map of broad emotion categories across the corpus (55). Although transformer-based models such as BERT or RoBERTa may offer stronger performance in some classification tasks, their outputs depend on model architecture, training data, domain fit, and classification thresholds. In this study, a lexicon-based approach was considered more appropriate because the quantitative outputs were used as broad contextual signals and were interpreted alongside the reflexive thematic analysis rather than as standalone measures. Future work could extend this design by comparing lexicon-based findings with transformer-based sentiment and emotion models.

Topic modelling was conducted using latent Dirichlet allocation (LDA) to identify recurring discursive clusters across the corpus. The model was specified with eight topics, unigrams and bigrams, a minimum document frequency of 5, a maximum document frequency of 0.6, and a fixed random state of 42. The eight-topic solution was retained on the basis of interpretability and its usefulness for mixed-methods integration rather than on formal coherence optimisation. The resulting topics were treated as exploratory semantic groupings rather than as qualitative themes or definitive latent structures.

Keyword co-occurrence network analysis was used as an exploratory supplement to the sentiment and topic analyses. In this network, nodes represented keywords and edges represented co-occurrence within the same comment. The analysis was used to identify recurrent semantic associations and the central concepts structuring public discourse on AI mental health support. Because some generic conversational terms remained in the network despite preprocessing, the network analysis was interpreted cautiously and used mainly to support patterns identified in the sentiment, topic, and thematic analyses.

#### Qualitative sampling and reflexive thematic analysis

For the qualitative phase, a purposive hierarchical sample of high-engagement comments was constructed from the cleaned corpus. Parent comments were selected on the basis of like count, and up to seven replies were retained for each selected parent comment in order to preserve conversational context. The qualitative sample focused on high engagement comments because the aim of the thematic analysis was to examine publicly resonant viewer discourse rather than to produce a statistically representative account of all comments. Likes and replies were treated as indicators of discursive salience, helping identify comments that other viewers had endorsed, challenged, or extended. This produced a final qualitative dataset of 460 comments for ATLAS.ti coding and reflexive thematic analysis. Collectively, these comments accounted for 164,932 likes, indicating that the qualitative sample captured a substantial share of the most publicly engaged discussion within the broader corpus.

The qualitative analysis was conducted using Braun and Clarke’s (56) reflexive thematic analysis (RTA). RTA was chosen because of its epistemological flexibility and its suitability for identifying patterns of shared meaning in digital discourse. Consistent with this approach, themes were treated as interpretive constructions developed through researcher engagement with the data, rather than as fixed entities waiting to be discovered.

Initial coding was supported by ATLAS.ti. However, all interpretive theme development remained researcher-led. Codes were reviewed, grouped, and refined across multiple analytic cycles, with reflexive memoing used to document interpretive decisions, uncertainties, and tensions throughout the process. Theme development was guided by the principle that themes should be interpretive rather than merely descriptive, theoretically meaningful, and grounded in recurring patterns across the dataset (56). Both authors engaged in ongoing reflexive dialogue during the analytic process, comparing interpretations and questioning emerging readings in order to strengthen the depth and coherence of the final thematic account.

#### Mixed-Methods integration

Quantitative and qualitative findings were integrated through systematic comparison and meta-inference (48). The computational analyses provided a corpus-level map of affective and discursive patterns, while the qualitative analysis explained how these patterns were constructed and negotiated by commenters. For example, the co-presence of trust and fear in the NRC analysis corresponded to the mixture of optimism, caution, and boundary-setting evident across the qualitative themes. Similarly, topic modelling and keyword analysis identified recurrent clusters around human therapists, replacement, need, and support, while reflexive thematic analysis clarified the meanings, tensions, and conditions embedded within those clusters. The two strands were integrated at the interpretation stage and informed the overall account developed in the Results and Discussion.

### Ethical considerations

This study analysed publicly available YouTube comments posted in a space with a reasonable expectation of broad public visibility (57, 58). Consistent with current internet research ethics guidance, formal ethical review was not required for the analysis of these publicly accessible data (59, 60). Ethical practice was nonetheless guided by the principles of situated ethics (61). Usernames and other direct identifiers were removed prior to analysis and are not reported in the paper. Given that AI for mental health is a sensitive topic, and that some commenters disclosed experiences of trauma, distress, and relational harm, particular care was taken to minimise traceability. Reported quotations were selected and presented using strategies such as paraphrasing and ‘ethical fabrication’ to reduce the likelihood of participant identification through online search engines (58, 62). To clarify the application of this practice in the present study, direct quotation was used where traceability risk was judged to be low. Paraphrasing was applied where comments contained identifying contextual details, such as specific geographic references, named individuals, or distinctive personal circumstances, that could increase the risk of traceability even without usernames. Ethical fabrication, in the sense described by Fiesler et al. (58) and Reagle (62), was used only where paraphrasing alone was considered insufficient to reduce identification risk, particularly where the combination of topic, phrasing, and platform context made a comment unusually searchable or identifiable. In all cases where modification was applied, the modified extract was checked against the original comment to ensure that the interpretive meaning and analytical function of the extract were preserved. This process was used to balance participant protection with analytic fidelity and reflexive transparency.

## Results

### Sentiment analysis

A total of 7,949 comments across ten YouTube videos were analysed using VADER sentiment analysis. Overall, the corpus leaned positive, with 57.93% of comments classified as positive, 24.58% as negative, and 17.49% as neutral. This suggests that public discourse on AI for mental health support was more supportive than resistant, although critical and sceptical responses remained substantial.

Sentiment varied by video title (see Table 2). *Therapist vs. Artificial Intelligence* showed the highest proportion of positive comments (72.6%), while lower positivity was observed for more sceptically framed videos, including *AI and mental health: Young people turning to AI therapist bots* (45.9%) and *AI-powered mental health chatbots developed as a therapy support tool* (45.1%). *Is ChatGPT therapy a horrible idea?* remained more mixed, with 50.5% positive comments despite its large comment volume. Overall, these patterns suggest that video framing was associated with the affective tenor of audience responses.

**Table 2:** Sentiment distribution by video title (VADER analysis; *N* = 7,949).

Title of the Video	n	Positive (%)	Negative (%)	Neutral (%)
AI and mental health: Young people turning to AI therapist bots | BBC News	172	45.9	29.1	25
AI-powered mental health chatbots developed as a therapy support tool | 60 Minutes	286	45.1	33.9	21
Can AI Really Help With Mental Health?	950	63.8	19.5	16.7
Can AI Replace Therapists? | Psychiatrist Explains	1,056	66.4	20.5	13.2
Can Chat GPT Replace Your Therapist?	504	62.1	24.8	13.1
Fixing My Brain with Automated Therapy	2,582	58.1	25.9	16
I tried an AI therapist. Does it actually work? | BBC News	219	49.8	34.2	16
Is ChatGPT therapy a horrible idea?	1,561	50.5	25.5	24
Therapist vs. Artificial Intelligence - I answer your questions #chatgpt #mentalhealth	234	72.6	16.2	11.1
What's scary about AI Therapy ft @TheDiaryOfACEO	385	54.8	26	19.2
**Total**	**7,949**	**57**.**93**	**24**.**58**	**17.49**

Percentages are rounded to one decimal place. Sentiment labels are assigned using VADER compound score thresholds (positive ≥0.05; negative ≤−0.05; neutral between −0.05 and 0.05).

### Emotion analysis

Emotion profiling was conducted using the NRC Emotion Lexicon. As shown in Table 3, positive emotion accounted for the largest share of all emotion hits (23.71%), followed by trust (14.98%) and anticipation (10.03%). Joy contributed a further 8.77%, indicating that the emotional tone of the corpus was not predominantly fearful or hostile.

**Table 3:** Emotion distribution across the corpus (NRC lexicon; *N* = 7,949 comments).

Emotion	Raw Count	% of all emotion hits
positive	17,646	23.71
trust	11,150	14.98
negative	9,765	13.12
anticipation	7,461	10.03
joy	6,526	8.77
fear	5,893	7.92
sadness	5,269	7.08
anger	4,474	6.01
surprise	3,178	4.27
disgust	3,059	4.11

Raw counts reflect total lexical matches across all comments. A single comment may contribute hits to multiple emotion categories. Percentages are calculated as a proportion of total emotion hits (74,421).

At the same time, negative affect remained substantial. The negative category accounted for 13.12% of all emotion hits, while fear (7.92%), sadness (7.08%), anger (6.01%), and disgust (4.11%) were also prominent. The coexistence of positive and trust-related language with fear and negative affect suggests that public responses to AI mental health support were characterised by cautious openness rather than simple acceptance or rejection. Surprise was the least frequent emotion category (4.27%), indicating that strong reactions were present, but were not primarily driven by shock or novelty.

### Topic modelling

LDA topic modelling produced an eight-topic solution (see Table 4). These topics are interpreted as exploratory lexical clusters rather than as qualitative themes. Topic 5 was the most prevalent (15.49%) and centred on terms such as need, human, feel, life, problems, and talk, suggesting discussion around personal mental health experiences and unmet support needs. Topic 4 was the second most prevalent (14.55%), organised around human, therapists, replace, connection, and social, indicating that the question of whether AI should replace human therapists was a major focus of the corpus. Together, these two topics accounted for 30.04% of all comments.

**Table 4:** LDA topic model: top words and prevalence (*N* = 7,949 comments).

Topic	Top words	% of comments	Interpretive label
5	need, better, it's, human, therapists, time, feel, life, real, good, person, problems, talk, want, problem	15.49	Personal mental health experience and emotional need
4	human, it's, therapists, replace, humans, dr, good, real, don't, connection, social, need, better, want, person	14.55	The human vs. AI replacement debate
1	want, it's, video, i'm, can't, don't, chat, going, i've, you're, that's, good, hear, work, actually	14.17	General AI chatbot engagement
7	love, videos, video, i'm, content, thank, man, jacob, feel, thanks, youtube, channel, it's, game, great	13.14	Creator appreciation and channel engagement
2	it's, app, time, i'm, don't, good, lot, ask, right, feel, pay, going, video, work, insurance	12.79	Practical and accessibility concerns
8	video, cbt, years, it's, talk, great, new, lot, i'm, feel, long, better, person, good, thank	11.02	CBT experiences and personal testimony
6	don, good, life, ve, person, work, chat, feel, does, men, apps, better, actually, ask, data	9.88	Mixed exploratory cluster
3	cbt, dan, algorithm, don't, comment, change, oh, yes, data, idea, it's, toomey, dan toomey, going, commenting	8.96	Platform meta-commentary and discourse fragmentation

Other topics reflected broader chatbot engagement (Topic 1, 14.17%), practical concerns about apps, cost, and access (Topic 2, 12.79%), and CBT-related experiences and personal testimony (Topic 8, 11.02%). Topic 6 was more fragmented and is treated cautiously as a mixed exploratory cluster rather than a clearly defined theme.

Topics 3 and 7 contained substantial content that was not directly focused on AI mental health support. Topic 7 (13.14%) was dominated by terms associated with creator appreciation and channel engagement, while Topic 3 (8.96%) captured platform meta-commentary, including recurring CBT acronym jokes and references to algorithmic content promotion. Rather than treating these topics as noise to be discarded, they are retained as evidence of the platform conditions shaping the corpus. Their presence indicates that YouTube comment sections are not purely deliberative spaces, but also include parasocial engagement, humour, and platform-specific interaction. The substantive AI mental health themes therefore emerged within a broader comment ecology shaped by both topical and non-topical engagement.

### Keyword co-occurrence network

Keyword co-occurrence network analysis was conducted as an exploratory supplement to the sentiment and topic analyses. The network comprised 2,527 nodes and 2,889 edges. The most prominent cluster was organised around terms such as human, therapists, replace, and CBT, suggesting that the discourse was strongly shaped by comparisons between AI support and human therapeutic care. The most frequent substantive co-occurring pairs were human–replace (29), replace–therapists (23), and human–therapists (22), indicating that the central issue in the corpus was whether AI should supplement or replace human therapists. Because some generic conversational terms remained in the network, this analysis is interpreted cautiously and used mainly to support patterns identified elsewhere in the data. A cleaned visualisation of the keyword co-occurrence network is provided in Supplementary Figure S1.

### Thematic analysis

Five interpretive themes were developed through repeated engagement with the coded corpus. Consistent with reflexive thematic analysis, each theme represented a pattern of shared meaning across multiple extracts rather than a simple aggregation of frequently occurring codes.

#### Theme 1: A bridge, not a destination

The dominant position in the corpus was neither uncritical acceptance nor outright rejection of AI mental health support. Commenters expressed a pragmatic, conditional endorsement grounded in the failures of existing systems. AI was evaluated not against a well-functioning therapeutic relationship, but against waiting lists, unaffordable fees, and the absence of any alternative. A commenter on *Fixing My Brain with Automated Therapy* (Quotation 1:336) captured this plainly: “It shouldn't be a replacement, it should be a supplement. Some people need daily support, but most people can't access a counselor on a daily basis. The resources simply aren't there.” Another located the same gap more bluntly (Quotation 1:214): “in the uk you just get put on the waiting list and never actually get any support. maybe the mental health system is broken all over.”

This framing extended beyond structural access to psychological safety. Several commenters described situations in which human support was technically available but emotionally inaccessible. One commenter (Quotation 1:218) wrote: “I do have actual people I could vent to but… I can't. And I just don't like talking about trauma to real people.” AI offered a space to disclose without the social risks associated with human interaction. The same dynamic was evident in comments describing acute crisis, where immediacy mattered more than clinical adequacy. A commenter who identified as a psychology student (Quotation 1:241) recalled: “these apps were my only resource for not killing myself when I was 14… being in genuine crisis and having nowhere else to turn,” while also acknowledging the “problems and sinister implications” of such apps. This coexistence of relief and critique was characteristic of the theme.

These comments do not amount to an endorsement of AI therapy as a sufficient solution. Rather, they point to the conditions that made AI necessary. The clearest formulation came from a commenter (Quotation 1:306) who wrote: “It's OK if it's the first ‘person’ you feel safe opening up to, just make sure it isn't also the last.” That distinction — AI as entry point rather than endpoint — captures the organising logic of the theme.

#### Theme 2: It says what you want to hear

A persistent concern across the corpus was that AI's apparent supportiveness was structurally compromised by its design orientation towards user approval. Commenters observed that AI systems tended to validate rather than challenge, to affirm rather than interrogate — and that this made them feel good in the short term while undermining the conditions necessary for genuine therapeutic progress. One commenter (Quotation 1:231) described this dynamic with unusual clarity: “AI is cheaper, faster, and will tell you everything you want to hear. It's a people pleaser. Plus, people can't see the significant downsides immediately, so they don't care. It feels good, so how can it be bad?” The concern here was not that AI was unhelpful, but that its helpfulness was fundamentally limited— comfortable, frictionless, and ultimately shallow.

This problem of sycophantic agreement was identified not only by sceptics but by commenters who reported using AI regularly and finding value in it. Several described active strategies to counteract AI's tendency to agree. One commenter (Quotation 1:318) wrote: “I also point out when I think it is being too nice.. there are many times that it says how rare my emotional maturity is. But I'm like ‘Is it really that rare? Or are you just massaging my ego?” Another (Quotation 1:316) described asking inverse questions and requesting AI to argue both sides, adding: “It is definitely trying to please you. It's your responsibility to continue probing instead of stopping once you've heard what you wanted to hear.” These comments reveal a sophisticated user awareness of AI's limitations — but they also reveal that managing those limitations required effort that many users were unlikely to invest.

The deeper concern was that validation without challenge may provide comfort without growth. One commenter (Quotation 1:252) drew a precise distinction: AI “can only validate what you say and in the best case scenario, offers some insights that only become emotional intelligence if you reevaluate it. It's like a diary that talks back and.. says all the right things aka things you want to hear.” A further commenter (Quotation 1:250) sharpened this: “I always think that AI therapy could make a person FEEL better, but i really doubt its ability to make a person function in their life better — which is the purpose of therapy.” The distinction between feeling better and functioning better was central to this theme. AI could produce emotional relief; it could not reliably produce the kind of growth that requires being questioned, challenged, or told something unwelcome.

#### Theme 3: the machine finds the predictable in the human

A recurring strand of commentary moved beyond scepticism about AI's effectiveness to a more fundamental claim: that AI was incapable, by design, of replicating the irreducible human element of genuine therapeutic encounter. The concern was not primarily technical. It was relational. Commenters argued that what makes therapy work — the capacity of another person to be genuinely moved, surprised, or troubled by what they hear — was precisely what AI could not simulate. One commenter (Quotation 1:278) who reported using both ChatGPT and a human therapist for a year wrote: “it still feels like talking to a plastic person, and not at all comparable to a real therapist. I would come to ChatGPT and my therapist with the same issues and the therapist by far knew the right questions to ask in order to change my perspective.” The word “plastic” recurred across the corpus as an intuitive descriptor for AI interaction — present in form, hollow in substance.

Several commenters located this hollowness in AI's inability to respond with authentic human reaction. One commenter (Quotation 1:349) described a pivotal moment in their own therapy: “One of the things that was best in therapy for me was my therapist's shock at my past, my therapist admitting in horror no child should ever have to experience what I did. The abject horror she had is what really started helping me heal.. It's one of the biggest reasons I feel AI could never truly replace therapy.” What this commenter identified was not a technical capability gap but an ontological one. AI cannot be horrified. It cannot be genuinely affected by what it is told, and that affective response — the therapist's authentic reaction — was itself therapeutic.

This argument was given its sharpest formulation in a comment (Quotation 1:201) that drew directly on the logic of the video it was responding to: “it doesn't find the human in the machine, it finds the predictable, repetitive machine in the human.” The phrase captures a critique that went beyond AI's limitations to question what AI-mediated therapy actually does to its users — not expanding their self-understanding, but reducing them to the patterns that a statistical model can process. A further commenter (Quotation 1:305) identified the same dynamic from the user side: “when talking to AI it always says something like ‘that is a very good question to root out the deepest questions in life, with courage and dignity’ and it feels so fake and sugar-coated.” The performance of empathy, without its substance, produced not comfort but distrust.

#### Theme 4: Social media 2.0

A distinct cluster of comments approached AI therapy not as an individual clinical question but as a societal one. These commenters drew explicit parallels between the adoption of AI and the earlier adoption of social media, arguing that both had been embraced without adequate reckoning with their structural consequences for human connection. The concern was not that AI would fail to help individuals, but that it would succeed well enough to displace the harder work of building and maintaining human relationships — deepening the loneliness epidemic it claimed to address. One commenter (Quotation 1:221) articulated this most fully: “My bigger concern is not that AI can replace humans in relationships, it is that people will insist they can and ignore the downsides of such a substitution. Like how social media substituted for human interaction in a way that initially seemed effective.. and led to a generation of lonely, socially inept people who really struggle in actual relationships.”

This comparison to social media functioned as a structural warning rather than a simple analogy. Commenters who invoked it were arguing that the problem with AI, as with social media, was not that it provided nothing — it was that it provided enough to reduce the incentive to seek something better. One commenter (Quotation 1:328) wrote: “As someone in IT our march towards AI is terrifying. It reminds me of our trek to social media — we touted it as this great revolution without analysing the negatives. Now we know that by and large social media isn't great for us and we're going to make the same mistakes with AI.” Another (Quotation 1:222) was more blunt: “AI could be the same problem as social media in that it makes humans less inclined to reach out and make more social connections with real humans. I am always worried we are getting too close to what humans were like in WALL-E.”

The loneliness concern was not abstract. Several commenters connected it to the specific conditions of AI use — people who were already isolated, already struggling to connect, turning to AI and thereby reducing further their exposure to the friction and reciprocity of human relationships. One commenter (Quotation 1:335) made the systemic stakes explicit: “Considering the fact that loneliness and lack of human connection is an epidemic in today's day and age, I think AI replacing therapists and more jobs is a terrible idea.” What this theme surfaces is a population-level concern that sits alongside the individual-level benefits identified in Theme 1. AI may help the individual who has no other option; it may simultaneously worsen the conditions that produce people with no other option.

#### Theme 5: who owns my pain?

The fifth theme concerned data, privacy, and the commercial infrastructure within which AI mental health tools operate. Commenters expressed unease — sometimes acute — about the implications of disclosing personal and psychological information to systems owned by corporations with commercial interests. This concern was sharpest in relation to what one commenter (Quotation 1:197) described as the inevitable consequence of sharing therapy data with AI: “Whatever OpenAI does with therapy data is gonna make the Cambridge Analytica scandal look like a leaflet campaign.” The comparison was hyperbolic, but the underlying concern was consistent across a broader set of comments: that the intimacy of psychological disclosure made data misuse in this domain categorically more serious than in others.

Several commenters identified a paradox in their own behaviour — or in the behaviour of people around them — that sharpened the critique. One commenter (Quotation 1:260) observed: “People getting upset over companies tracking and selling their data while sharing intimate thoughts and confessions with a software is a hilariously depressing paradox.” Another (Quotation 1:353) was more direct: “Are you really going to tell an app your deepest darkest concerns? Users should be terrified of what their data will be used for.” These comments did not argue that people were wrong to use AI for emotional support. They argued that the conditions of that use — corporate ownership, data retention, and the absence of protections equivalent to clinical confidentiality — were poorly understood by most users and inadequately addressed by most platforms.

The concern extended to the possibility of commercial capture of the therapeutic relationship itself. One commenter (Quotation 1:213) imagined a near-future scenario with dry precision: “I cant wait to say something to my ai therapist and get a response guided by nothing but sheer product placement.” A further commenter (Quotation 1:311) drew the boundary at the level of personal choice: “I don't want to willfully train chat gpt with my trauma.” The phrasing is analytically significant. The commenter did not say they feared data misuse in the abstract. They objected to the specific act of contributing their psychological experience as training material for a commercial system — framing disclosure not as therapy but as unpaid labour. Taken together, these comments construct AI mental health tools not simply as potentially helpful or potentially harmful, but as sites where personal vulnerability intersects with commercial interest in ways that existing regulatory frameworks have not yet resolved.

### Integration of quantitative and qualitative findings

The quantitative and qualitative findings converged on a consistent picture. Sentiment was moderately positive overall (57.93%), but this masked important variation. The qualitative analysis showed that positive sentiment was rarely unconditional. Instead, it was often tied to structural absence, crisis circumstances, or carefully bounded use, as captured in Theme 1. The high trust score (14.98%) and persistent fear score (7.92%) in the NRC analysis also reflected the ambivalence visible across all five themes, where commenters acknowledged AI's usefulness while also identifying clear limitations.

The keyword network and topic model supported these patterns in broad terms. The strongest co-occurring keyword pair, human–replace, reflected the central tension visible in Themes 3 and 4. Topic 4 (14.55%), centred on human, therapists, replace, and connection, aligned with concerns about empathy, relational depth, and the possible displacement of human care. Topic 5 (15.49%), centred on need, feel, life, and problems, corresponded closely to Theme 1's emphasis on unmet support needs and crisis-time use. The privacy concerns foregrounded in Theme 5 found more limited quantitative support in the prominence of data as a network node linking otherwise separate strands of discussion.

Where the methods differed, the qualitative analysis provided explanatory depth that the computational analysis could not. Sentiment analysis could identify broad positivity, but not the conditional logic within it. Topic modelling could identify recurring discursive clusters, but not distinguish between cautious acceptance and wholehearted endorsement. Reflexive thematic analysis made those distinctions visible.

## Discussion

Public responses to AI mental health tools in this study were neither simply positive nor negative. They depended on context. AI was viewed more favourably when human support was absent, delayed, too expensive, or emotionally difficult to access, and more critically when commenters compared it with what they believed good therapy should offer. The overall positive sentiment in the corpus (57.93%) therefore should not be read as straightforward endorsement. Much of that positivity was conditional, bounded, and shaped by structural gaps in care. This extends affordance theory beyond a narrow focus on perceived usefulness. Bucher and Helmond (44) describe affordances as relational possibilities that emerge through interactions between users and platforms. In this study, those affordances were judged not only in relation to the technology itself, but also in relation to the failures of the wider mental health system. AI appeared useful partly because existing care often did not. This interpretation is consistent with Russell and Bode's (63) analysis of AI mental health discourse on X, formerly Twitter. They similarly show that AI is often framed as a pragmatic but imperfect response to gaps in traditional care, while warning that such use may allow AI to become a technological patch for systemic problems rather than a prompt for structural reform.

This compensatory role of AI is consistent with earlier work, but the present findings sharpen it. Cross et al. (22) and Luo et al. (14) show that users value accessibility and availability, and Siddals et al. (6) note that AI can be useful for immediate emotional support. The comments in this study push that point further. Many users described turning to AI at moments of acute need, including long waits for care, high therapy costs, and situations where speaking to a real person felt too difficult. For some, AI was not a supplement to therapy. It was the only support they felt they had. That is an uncomfortable finding. It suggests that the strongest case for AI in mental health may lie less in its therapeutic strength than in the weakness of the systems around it. At the same time, this point should be interpreted carefully. People who describe crisis-related AI use in YouTube comments are self-selected, and these data cannot tell us how common such experiences are among the broader population of users.

The study also shows that many users do not trust AI uncritically, even when they find it helpful. They are aware that these systems often flatter, validate, and agree. Some commenters described active strategies for managing this, such as asking the model to challenge them, posing the same issue from different angles, or deliberately questioning responses that felt too comforting. This makes the usual contrast between algorithm aversion and algorithm appreciation look too simple. People may prefer algorithmic judgement in some settings, while Hoff and Bashir (46) show that they may reject it when errors become visible. The present findings suggest something more mixed. Users may appreciate AI and distrust it at the same time. Haensch (16) reports similar workaround behaviour in social media discourse on AI therapy, and Jung et al. (17) show that users actively manage disclosure when speaking to large language models. This study adds that such management is not occasional or trivial. It is ongoing work. That matters because the users most in need of support may also be the least able to do that work consistently. Taken together, these patterns extend the algorithm aversion and appreciation framework beyond a simple distinction between trust and distrust. In this study, users appeared to engage with AI dynamically and strategically, relying on its availability while also testing, questioning, and managing its limitations.

The clearest limitation of AI in this study was not technical but relational. Commenters repeatedly said that AI could sound supportive without feeling real. They drew attention to things they believed only a human therapist could offer: genuine surprise, moral recognition, emotional presence, and the ability to ask difficult questions in a way that changes perspective. This complicates some of the more optimistic work on digital therapeutic alliance. Beatty et al. (28) and Schäfer et al. (29) report alliance scores for AI tools that look promising, and Darcy et al. (9) describe strong bonds between users and conversational agents. The present findings do not necessarily contradict those studies, but they do suggest that standard alliance measures may miss something important. Commenters in this dataset often described the most powerful moments in therapy as moments when another human being was visibly affected by what they heard. Wampold and Flückiger (8) argue that the therapeutic bond is central to psychotherapy. The comments here suggest that users understand that bond in deeply human terms. What they felt was missing from AI was not just skill, but human presence itself. That does not mean AI cannot help. It does mean that many users still see a line between support that feels useful and support that feels genuinely therapeutic.

The comparison with social media is also important. Many commenters argued that AI might follow the same path: widely adopted because it seems useful, then later recognised as harmful in ways that were not obvious at first. Turkle (45) has made a related argument about digital communication more broadly, suggesting that technologies can make connection easier while also reducing people's tolerance for the demands of real relationships. That concern appeared clearly in the dataset. Commenters worried that AI might not only respond to loneliness, but also deepen it by making human connection easier to avoid. This is one of the most important points raised by the study. Even if AI reduces distress in the short term, it may still have longer-term social costs that current evidence cannot capture well. Lee et al. (15) and Cross et al. (22) identify similar concerns in survey-based work. What this study adds is evidence that ordinary users are already thinking about these issues in sophisticated ways. A major gap in the literature remains the absence of longitudinal research on whether regular use of AI mental health tools changes how people relate to other human beings over time. Turkle’s (45) social displacement framework, applied here to AI mental health tools, therefore raises a specific empirical question: whether repeated reliance on AI for emotional support may, over time, reduce opportunities for more demanding forms of human interaction. This remains underexamined in the context of AI mental health support.

The privacy findings point in a similar direction. Commenters were not only worried about abstract data breaches. Many seemed to understand that deeply personal disclosures made to AI systems could become part of commercial systems that were not built primarily for care. This is where Zuboff’s (47) idea of surveillance capitalism becomes useful. Her argument is that personal experience is increasingly turned into behavioural data that can be stored, analysed, and used for profit. The commenters in this study often described this in plain, direct language. They worried that emotional pain, trauma, and intimate disclosure might become data for systems they did not control. Rahsepar Meadi et al. (11) document ethical concerns around conversational AI in mental health care, and Benda et al. (12) note legal and accountability problems. The present findings add that users themselves are already alert to these risks. This matters because it suggests that distrust is not simply a design problem. It may reflect a rational reading of the political economy within which these tools operate.

Three practical implications follow from these findings. First, AI mental health tools should be judged in relation to the real access conditions of the people using them. It is not enough to ask whether they are as good as therapy in principle. We also need to ask what alternatives users actually have. Second, the tendency of these systems to validate and please should be treated as a serious design issue. A tool that comforts without ever challenging may feel good while still limiting reflection, growth, or change. Third, privacy and governance should be treated as central clinical concerns, not secondary technical ones. If users are sharing highly sensitive psychological material with commercial systems, then protections should reflect that reality. The comments in this study suggest that users already understand the stakes. Policy and design have not yet caught up.

## Limitations

Several limitations should be noted. First, YouTube comments are not demographically representative of broader public attitudes toward AI mental health tools. Commenters are self-selected and may be more digitally engaged, informed, or opinionated than the wider population. The videos analysed in this study may also have attracted viewers with prior interest in mental health, therapy, technology, or AI. Some viewers may have been mental health professionals, students, researchers, or people with personal experience of mental health support, although this cannot be confirmed from the available data. For this reason, the findings should be understood as viewer discourse around these specific YouTube videos, rather than as evidence of general population attitudes. The results should therefore not be generalised beyond this context without further research.

Second, the data were shaped by the YouTube platform and by the search process used to identify videos. YouTube's search and recommendation systems may influence which videos become visible to researchers and viewers and which discussions receive more engagement. Search results may also vary depending on factors such as location, browsing history, device, timing, and platform updates. In addition, each video was produced by a specific creator or channel, and the framing of the video and its channel audience culture may have influenced the tone and content of the comments. For this reason, the ten videos analysed here should be understood as a purposive sample of English language YouTube discourse around AI mental health support, not as a comprehensive or neutral sample of all online discussion on the topic. The sentiment analysis also showed that videos with a more sceptical framing tended to receive more critical comments.

Third, the study included only English language videos and comments. This limits the cultural and linguistic range of the findings. Discussions of AI mental health support in other languages and cultural contexts may show different concerns, emotions, and interpretations.

Fourth, the computational tools have known limitations. VADER may misclassify sarcasm, irony, and highly contextual language, while the NRC lexicon captures lexical emotion cues but cannot distinguish between describing an emotion and experiencing it. The study did not include a separate manually coded validation subset for the VADER or NRC outputs. The LDA model was also used as an exploratory tool and was not optimised through formal coherence metrics such as Cv. For these reasons, the sentiment, emotion, and topic modelling findings should be interpreted as descriptive corpus-level indicators rather than precise classifications of individual comments or definitive representations of latent discourse structure. These outputs were not used as standalone evidence, but were interpreted alongside the qualitative findings to support mixed-methods integration.

Fifth, the qualitative analysis was based on the portion of the ATLAS.ti quotation export in which full comment text was retrievable. Although this material was extensive and sufficient for theme development, some earlier highly engaged comments could not be directly quoted. The use of high engagement comments may have introduced popularity bias. Comments that are emotionally vivid, polarising, humorous, or strongly worded may attract more likes and replies than quieter, more ambivalent, or more moderate comments. For this reason, the thematic findings should be interpreted as patterns within publicly resonant YouTube discourse, rather than as a full representation of all viewer perspectives.

Finally, reflexive thematic analysis is interpretive by design. The themes presented here reflect one analytically grounded reading of the data rather than the only possible interpretation. The theoretical framing adopted in this study may also have foregrounded structural and relational concerns more strongly than other possible dimensions, such as individual differences in AI readiness or cultural variation in attitudes towards technology-mediated care.

## Conclusion

Public engagement with AI mental health tools in this study was marked by conditional rather than absolute acceptance. AI was valued where it filled a gap left by the mental health system, but resisted where it appeared to threaten the relational depth, authenticity, or privacy that users continued to regard as central to genuine care. These findings shift the terms of the debate. The key question is not simply whether AI mental health tools work, but under what conditions, for whom, and with what trade-offs. The implications are practical. Governance frameworks need to extend stronger protections to AI wellness tools that handle sensitive psychological disclosure. Developers need to design not only for comfort, but also for challenge and responsible boundary-setting. Researchers, in turn, need longitudinal evidence on whether AI mental health use supports, reshapes, or displaces engagement with human care.

## Data Availability

Publicly available datasets were analyzed in this study. The original data were collected via the YouTube Data API v3 (https://developers.google.com/youtube/v3). The dataset is available from the corresponding author upon reasonable request.
