# Supplementary material for: A bridge, not a destination: YouTube viewer perspectives on AI mental health support and human therapy
Source: Front Digit Health. 2026 Jun 3;8:1851632. doi: 10.3389/fdgth.2026.1851632 (PMC13271906; doi:10.3389/fdgth.2026.1851632)
Supplement: Supplementary file 1 [file Image1.pdf]

Supplementary Figure S1 *Cleaned keyword co-occurrence network in YouTube comments*

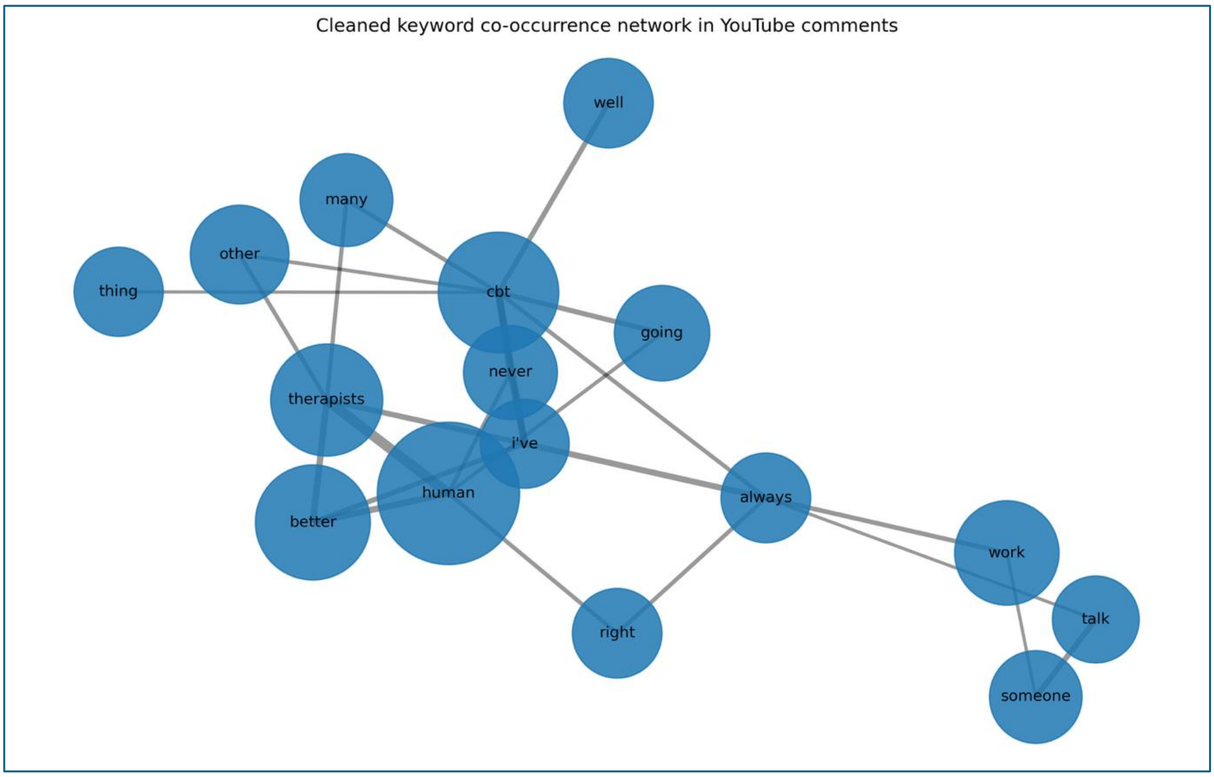

Node size reflects keyword frequency, and edge thickness reflects co-occurrence frequency within the same comment. The figure is presented as an exploratory visual supplement to the keyword network analysis.
